# Inflammation-related proteomics demonstrate landscape of fracture blister fluid in patients with acute compartment syndrome

**DOI:** 10.3389/fimmu.2023.1161479

**Published:** 2023-04-06

**Authors:** Yubin Long, Yiran Li, Tao Wang, Andrew Ni, Jialiang Guo, Qi Dong, Shuo Yang, Junfei Guo, Ling Wang, Zhiyong Hou

**Affiliations:** ^1^ Department of Orthopaedics, The Third Hospital of Hebei Medical University, Shijiazhuang, China; ^2^ Country Department of Orthopaedic Surgery, Baoding No. 1 Central Hospital, Baoding, China; ^3^ Warren Alpert Medical School, Brown University, Providence, RI, United States; ^4^ The School of Medicine, Nankai University, Tianjin, China

**Keywords:** acute compartment syndrome, fractures, blisters, proteomics, cytokines, inflammation

## Abstract

**Background:**

Blisters are tense vesicles or bullae that arise on swollen skin and are found in a wide range of injuries. As a complication of fracture, fracture blisters are considered soft tissue injuries, which often lead to adverse effects such as prolonged preoperative waiting time and increased risk of surgical site infection. However, our previous study found that in patients with acute compartment syndrome, fracture blisters may be a form of compartment pressure release, but the specific mechanism has not been revealed. Here, we mapped out the proteomic landscape of fracture blister fluid for the first time and compared its expression profile to cupping and burn blisters.

**Methods:**

First, fluid samples were collected from 15 patients with fracture blisters, 7 patients with cupping blisters, and 9 patients with burn blisters. Then, the expression levels of 92 inflammatory proteins were measured using the Olink Target 96 Inflammation panel. Protein profiles were compared across the three groups using Differential Protein Expression Analysis and Principal Component Analysis (PCA).

**Results:**

Fracture blisters had significantly higher levels of 50 proteins in comparison to cupping and 26 proteins in comparison to burn blisters. Notably, PCA showed fracture blisters closely resembled the protein expression profile of burn blisters but were distinct from the protein expression profile of cupping blisters.

**Conclusion:**

Our study provides the first characterization of fracture blister fluid using proteomics, which provides a valuable reference for further analysis of the difference between blisters caused by fractures and those caused by other pathogenic factors. This compendium of proteomic data provides valuable insights and a rich resource to better understand fracture blisters.

## 1 Introduction

Acute Compartment Syndrome (ACS) is an orthopedic emergency that commonly occurs when a severe lower limb fracture causes increased pressure within a muscle compartment that compromises the surrounding tissue. Fracture blisters (FBs) typically form in conjunction with ACS, and Guo (1) previously found that the formation of blisters results in a significant decrease in compartment pressure. However, the mechanism of FBs formation underlying this decrease in pressure has not yet been elucidated. FBs have been defined as “skin bullae and blisters representing areas of epidermal necrosis with separation of the stratified squamous cell layer from the underlying vascular dermal layer by edema fluid” (2). FBs typically arise after high-energy orthopedic trauma to areas of the body, such as the ankle, wrist, elbow, and foot, where skin adheres tightly to the bone with little subcutaneous fat cushioning (3). They present similar to second-degree burns and can be either blood-filled or serous-filled (4). Although FBs are a somewhat uncommon complication of acute fracture injuries, occurring in only 2.9% of cases requiring hospitalization, they present significant challenges to treatment and management (5) and are often associated with increased infection rates and wound breakdown.

Recently, studies have turned towards proteomics to reveal mechanistic insights for many injuries including various blister fluids. In particular, Liu et al. compared protein expression in cupping and scald patients, finding that the two groups expressed significantly different proteins relating to the activation of immune pathways (6). Identification of differentially expressed proteins in the blister fluid provided clues to the potential regenerative function of cupping and suggests blister fluid is a suitable matrix to explore biological pathways of disease and to act as a powerful biomarker. In addition, Zang et al. suggest that blister fluid is a viable matrix for burn injury research, as it can reflect both systemic and local micro-environmental responses. More than 600 proteins were quantified in 87 blister fluid samples from pediatric burn patients. These data were correlated with burn depth and healing time, allowing classification of burn wound severity and assisting with clinical decision-making (7). Similarly, Solimani et al. utilized liquid chromatography with tandem mass spectrometry to characterize the bullous pemphigoid blister fluid proteome (8). Blister fluid was a valuable biologic resource, as it provided insight into both systemic and local microenvironment responses, allowing identification of several notable features unique to bullous pemphigoid.

Therefore, protein expression could be used to understand FB fluid function better, both by highlighting differences with other types of blister fluids and by yielding a valuable source of biomarkers. Here, we quantified the expression of 92 inflammatory proteins in the blister fluid of fracture, burn, and cupping patients. In particular, we aimed to use protein expression to identify differences between the blister fluids of the three groups. To do this, we performed Differential Protein Expression Analysis and Principal Component Analysis. This study further advances the understanding of molecular mechanisms underlying FBs and provides the first proteomic comparison of blister fluids in fracture, burn, and cupping patients.

## 2 Materials and methods

### 2.1 Subjects

This research was conducted at The Third Hospital of Hebei Medical University from September 2021 to April 2022. Samples were taken from three patient cohorts representing the fracture, burn, and cupping groups. First, serous-filled blister fluid samples were collected from 15 patients who had a lower leg fracture associated with ACS that required surgical treatment in the Department of Trauma Emergency. Second, blister fluid samples were collected from 9 patients in the Department of Burn and Plastic Surgery who had suffered burns. Finally, blister fluid samples were taken from 7 healthy adult volunteers who had cupping therapy.

The patients ranged in age from 20 to 68 years old and included both males and females. The ethical committee at Hebei Medical University’s Third Hospital reviewed and approved this study. The clinical trial number of the research was NCT04529330. The inclusion criteria were fracture patients with acute compartment osteofascial syndrome who were 18 years or older and developed FBs. Patients suffering from the following conditions were not eligible. The exclusion criteria included (1): serious comorbid conditions (e.g., life-threatening conditions or severe neurological defects); (2) patients who are unable to communicate reliably with the investigator or are unlikely to follow trial instructions; (3) severe infection; and (4) pregnancy.

### 2.2 Blister fluid extraction

Blister fluid was extracted for all patients as follows: the blister area was disinfected with iodophor, the blister fluid was extracted with a 5ml syringe and stored in an ice box, and the blister skin was retained for all patients. The blister was then disinfected with iodophor once more, and the outside was covered with a clean sterile dressing. For 15 minutes, samples were centrifuged at 2000rpm/mL. The supernatant was collected, and the blister fluid was pipetted into 2-3 bottles (400ul/bottle) and stored at -80°C until analysis. Blister fluid was collected from patients in the fracture and burn groups before appropriate treatment. Patients in the cupping group underwent dry cupping therapy using glass pots (5cm in diameter) for 10 minutes.

### 2.3 Protein extraction and cytokine measurement

Protein levels were quantified using the Olink^®^ target 96 Inflammation panel (Olink Proteomics AB, Uppsala, Sweden) according to the manufacturer’s instructions. The Olink panel is based on a Proximity Extension Assay (PEA) technology which has been well described (9). PEA allows for the simultaneous analysis of 92 analytes with 1 µL of each sample. In brief, oligonucleotide-labeled antibody probe pairs are allowed to bind to their target proteins in the samples. When two antibodies bind closely, the DNA oligonucleotides hybridize and are extended by DNA polymerization to form a polymerase chain reaction (PCR) reporter sequence. The sequences are detected, amplified, and quantified using a microfluidic real-time PCR instrument (Signature Q100, LC-Bio Technology CO., Ltd., Hangzhou, China). A list of all inflammatory proteins can be found in **Supplementary Table 1**. Protein abundance is reported as Normalized Protein Expression (NPX), an arbitrary unit in Log2 scale. A high NPX value indicates a high protein concentration. However, NPX values cannot be compared between different proteins.

Data quality control (QC) was carried out in two steps: First, the standard deviation was determined for each run for the detection control, incubation controls, and both. Only runs that had a standard deviation for each control less than 0.2 passed quality control. Second, the detection control and incubation control 2 were used to perform quality checks on each sample. All samples within the run were compared to the calculated run median of each of the controls. Samples failed the QC and were issued a QC warning in the data output file if they deviated more than 0.3 NPX from the plate median in relation to these two controls. All samples were measured successfully, and the number of quality control warnings was 6.06%. Of the 33 samples, samples from 2 patients in the burn group were excluded due to a quality control warning by Olink (i.e. 2 of the 11 burn patients were excluded for QC)

### 2.4 Statistical analysis

The R package “OlinkAnalyze” was used to identify differentially expressed proteins (DEPs) between two or three groups. A p-value below 0.05 was considered significant. Differential expression analyses were performed with a Welch 2-sample t-test (paired t-test) when performing pairwise comparisons and an Analysis of Variance (ANOVA) when comparing all three groups. This was corrected for multiple testing by the Benjamini and Hochberg method. Pair-wise differences were computed between all three groups (Fracture-Burn, Fracture-Cupping, and Burn-Cupping).

Principal component analysis (PCA) was performed with the “princomp” function in R (http://www.r-project.org/). PCA is a statistical procedure that converts hundreds of thousands of correlated variables (protein expression) into a set of values of linearly uncorrelated variables called principal components. PCA is well known to highlight the most important aspects of data variability and de-emphasizes the others. In particular, the dimensionality of the data is reduced so that it can be visualized in a few principal components where the most valuable information of the variables is retained. This enables the identification of sample clusters, for example, based on blister type, and the discovery of variables that drive this separation.

## 3 Result

### 3.1 Subject characteristics and clinical symptoms

**Figure 1:**
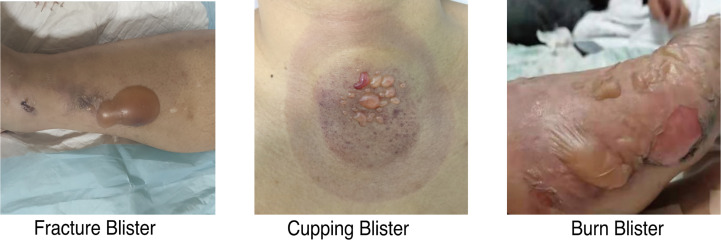
Images of blisters from fracture, burn, and cupping patients. Fracture blisters appear 24 to 48 hours after the fracture injury, and their exterior is similar to burn blisters and cupping blisters.

Thirty-one eligible participants provided clinical symptoms and contributed blister fluid samples. Blister fluid samples were collected from 15 fracture, 9 burn, and 7 cupping patients. Patient characteristics for the total study sample (n=31) were presented in **Table 1** by blister type. There was no statistical difference in age, sex, BMI, smoking, or any comorbidities. There was a significant difference in pain levels across the three groups and a significant difference in collection time as cupping samples were taken immediately after treatment. **Figure 1** characterized the appearance of blisters from patients in the three different groups, fracture blister group (FBG), burn blister group (BBG), and cupping blister group (CBG). Although all three types of blisters appeared on the skin surface, both FBs and BBs appeared as large blisters on the skin surface individually; on the other hand, cupping blisters appeared as multiple small, independent blisters scattered in the cupping area (**Figure 1**).

**Table 1:** Patient characteristics.

Variables	Fracture (N = 15)	Burn (N = 9)	Cupping (N = 7)	P-value
Age, mean (SD)	47.1 (13.4)	47.1 (11.0)	46.4(16.5)	0.994
Sex (Male/Female)	10/5	6/3	4/3	0.898
BMI (kg/m2)	23.4 (22.1–24.9)	23 (21.1–24.2)	23.9 (22.4–25.4)	0.63
Smokers, n	4	3	3	0.881
Comorbidities, n (%)				
Hypertension	4 (26.7)	3 (33.3)	2 (28.6)	1
Diabetes mellitus	3(20.0)	2 (22.2)	1(14.3)	1
Coronary heart disease	2 (13.3)	1(11.1)	1 (14.3)	1
Chronic obstructive pulmonary disease	1(6.7)	0 (0.0)	1 (14.3)	0.71
No comorbidity reported	9 (60.0)	5 (55.6)	5 (71.4)	0.896
Hours between injury and sample collection, mean (SD)	12.3 (4.2)	10.0 (5.1)	0.7 (0.2)	<0.001
Missing, n	0	1	0	–
VAS Score, mean (SD)	5.2 (1.1)	7.0 (0.9)	2.9 (0.7)	<0.001

### 3.2 Large overlap in detectable inflammatory proteins and distinct expression profiles

**Figure 2:**
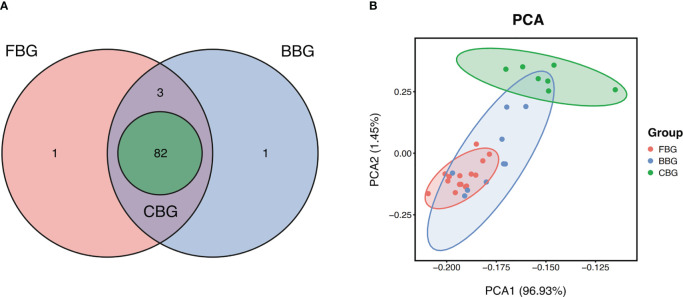
FBG, BBG, and CBG have unique protein expression profiles. **(A)** Venn Diagram shows the number of proteins with expression higher than the limit of detection shared and unique in FBG, BBG, and CBG. **(B)** PCA plot projects all 31 patients to two dimensions showing 3 groups differentiated by color. Each point represents a single patient, with patients of similar protein expression profiles positioned next to each other. Explained Variance: PCA1 = 96.93%, PCA2 = 1.45%.

In our PEA analysis, 86 proteins were higher than the Limit of Detection (LOD) in FBs, 86 proteins were higher than LOD in burns, and 82 proteins were higher than LOD in cupping. We considered a protein to be detectable if the number of samples with protein expression above LOD accounted for more than 75% of the total number of samples. All of the 82 proteins detected in cupping blisters could be detected in FBs and BBs. However, both FBG and BBG contained uniquely detectable proteins. IL-22RA1, IL-24, and NRTN were detected in both FBG and BBG but not CBG. In addition, TSLP was only detected in FBG while IL5 was only detected in BBG (**Figure 2A**). In total, 11 proteins were not detectable in any of the three groups.

Protein profiles of the patients were further investigated using Principal Component Analysis (PCA) to identify how similar the three types of blisters were. The PCA plot showed correlations between samples with points closer in space representing blister fluid samples with similar protein expression profiles. The FBG, BBG, and CBG clusters separated relatively well but still overlapped (**Figure 2B**). Most notably, CBG was the most different out of the three groups while FBG and BBG were quite similar. This suggested FB fluid and burn blister fluid are similar to each other but different from cupping blister fluid. In terms of variance, 96.93% was explained by the first component, and 1.45% was explained by the second component. This meant a single component is adequate to capture a majority of the information across the three groups.

### 3.3 Group differences in fracture, burn, and cupping patients’ level of inflammatory proteins

**Figure 3:**
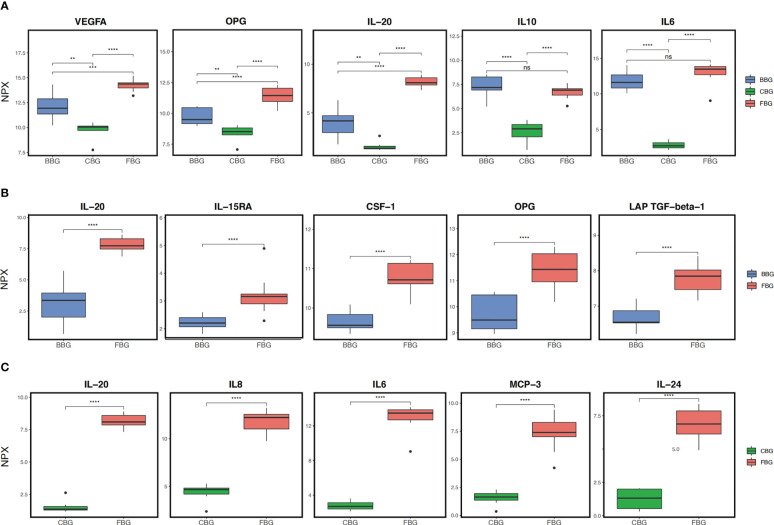
Comparison of protein expression in the three groups. **(A)** Box plots show the top 5 differentially expressed proteins across all three groups as identified by ANOVA. **(B)** Box plots show the top 5 differentially expressed proteins between FBG and BBG as identified by t-test. **(C)** Box plots show the top 5 differentially expressed proteins between FBG and CBG as identified by t-test. **p<0.01; *** p<0.001; **** p<0.0001; ns not significant.

First, we compared protein expression across all three groups. According to ANOVA, 56 proteins showed significant differences in expression between FBG, CBG, and BBG (**Table S1**). The top five of these proteins according to smallest P-value were IL6, IL-20, IL10, VEGFA, and OPG (**Figure 3A**). VEGFA, OPG, and IL20 were significantly different between all three groups with low expression in CBG, medium expression in BBG, and high expression in FBG. On the other hand, the difference in IL10 and IL6 expression in FBG and BBG was not significant. Instead of a gradual increase in expression from CBG to BBG to FBG, IL10 and IL6 had low expression in CBG and high expression in both FBG and BBG. In other words, two expression patterns were observed: (1) low CBG, medium BBG, high FBG or (2) low CBG, high BBG, high FBG.

**Figure 4:**
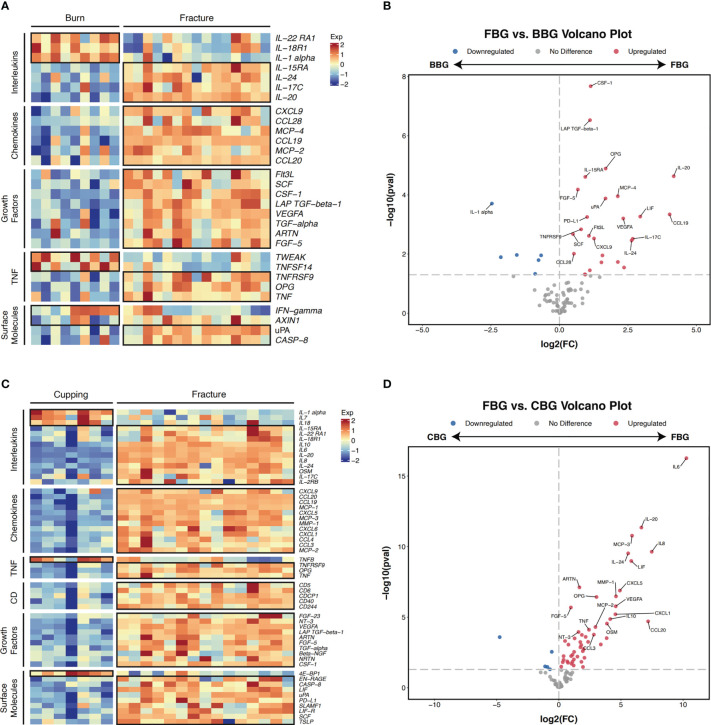
Pairwise comparison of differentially expressed genes. **(A)** Heatmap of differentially expressed proteins in FBG and BBG patients (P-value < 0.05). Proteins are manually annotated by protein family. **(B)** Volcano plot depicts differentially expressed genes between FBG and BBG patients. The gray dashed line indicates an exploratory cutoff of P-value < 0.05. Each red dot corresponds with a gene passing the exploratory cutoff. Genes of interest are marked with gene names. **(C)** Heatmap of differentially expressed proteins in FBG and CBG patients (P-value < 0.05). Proteins are manually annotated by protein family. **(D)**Volcano plot depicts differentially expressed genes between FBG and CBG patients. The gray dashed line indicates an exploratory cutoff of P-value < 0.05. Each red dot corresponds with a gene passing the exploratory cutoff. Genes of interest are marked with gene names.

Next, we performed pairwise comparisons between groups. Comparing FBG and BBG, we found significant differences in the expression of 32 proteins (**Figures 4A, B**) with 26 over expressed in FBG and 6 over expressed in BBG (**Figure 4B**). Interestingly, all proteins from the chemokine and growth factor families were significantly highly expressed in FBG compared to BBG (**Figure 4A**). The top five differentially expressed proteins between FBG and BBG according to smallest P-value were CSF-1, LAP TGF-beta-1, OPG, IL-20, and IL-15RA (**Figure 3B**). Then, a comparison of FBG and CBG showed significant differences in the expression of 55 proteins (**Figures 4C, D**) with 50 highly expressed in FBG and 5 highly expressed in CBG (**Figure 4D**). Similarly to above, all proteins from the chemokine, growth factor, and cluster of differentiation (CD) families were significantly over expressed in FBG compared to CBG (**Figure 4C**). The top five differentially expressed proteins between FBG and CBG were IL6, IL-20, MCP-3, IL8, and IL-24 (**Figure 3C**). Overall, the expression level of inflammatory proteins showed a considerable degree of heterogeneity and heavily favored over expression in FBG (**Figures 4B, D**).

### 3.4 Protein correlations in FBG reveal key protein-protein interactions

**Figure 5:**
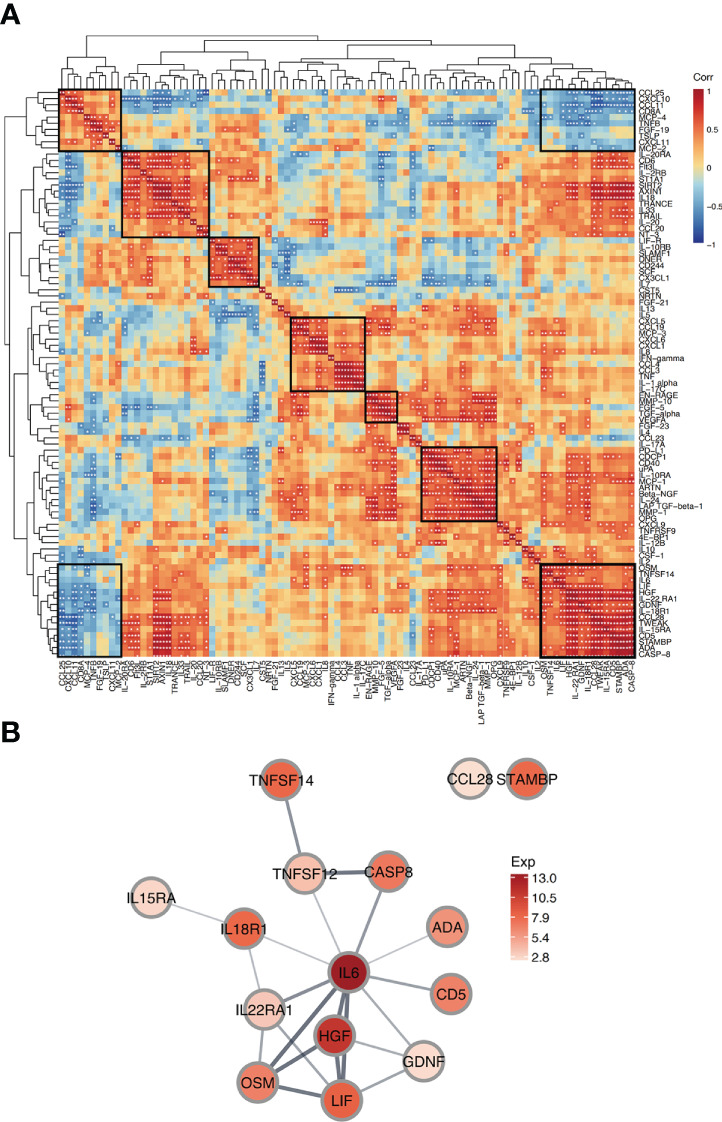
Correlation and interaction of proteins in FBG. **(A)** Correlation Heatmap depicts pairwise Pearson correlation coefficient values between proteins in FBG. Protein modules with high intercorrelation are marked with boxes. The rows and columns are grouped by hierarchical clustering. *p<0.05; **p<0.01. **(B)** Protein-protein interaction network shows predicted interactions between a group of proteins. Node colors represent average protein expression in FBG and edge weights indicate confidence level (strength of data support for the interaction).

To explore the relationship between proteins in FBs, a correlation analysis was performed for all proteins in FBG. Correlation coefficients (r) between proteins expressions in FB patients are shown in the correlation heatmap (**Figure 5A**). The 92 proteins had different levels of correlation with one another with some having inverse relationships. We identified several modules of proteins with a high degree of intercorrelation. For example, we saw strong correlations in the lower right module among IL-6, LIF, HGF, IL−22 RA1, GDNF, IL−18R1, CCL28, TWEAK, IL−15RA, CD5, STAMBP, ADA, and CASP−8 proteins (**Figure 5B**). Interestingly, this module was inversely correlated with the top left module which had strong correlations between CCL25, CXCL10, CCL11, CD8A, MCP−4, TNFB, FGF−19, TSLP, CXCL11, and MCP−2 proteins.

Next, to further explore one of these protein correlation modules, we created a Protein-Protein Interaction (PPI) network for a module with strong intercorrelations (**Figure 5B**). The network indicates the predicted associations for a particular group of proteins. In the PPI network, IL6 appeared as a central hub protein as it has connections with 10 other proteins. Due to the high connectivity of IL6, this protein likely played an important role in FB fluid. In particular, IL6 was most strongly connected with OSM, LIF, and HGF. These 4 proteins were all strongly interconnected and highly expressed in FBG, suggesting that they may work together mechanistically in FB fluid.

### 3.5 ELISA validation of TNF, TGF-beta-1, VEGFA and CASP-8 in FBG, BBG and CBG

**Figure 6:**
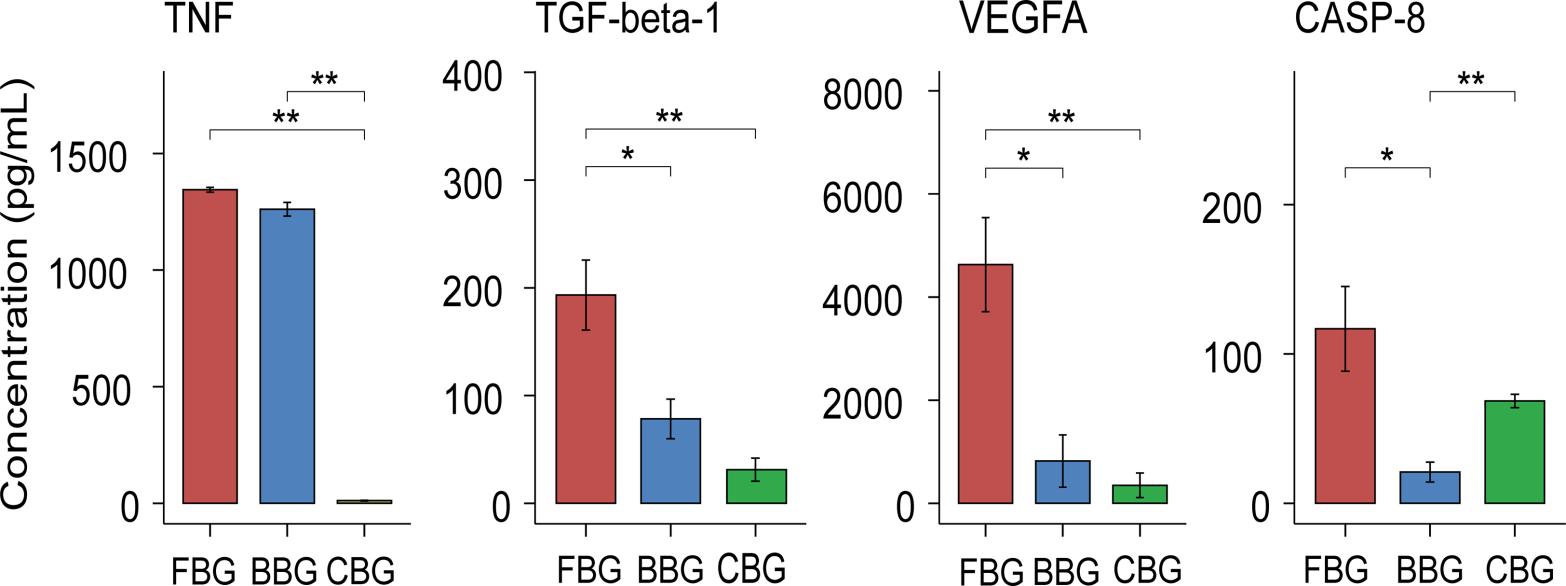
Validation of levels of inflammatory cytokines in blister fluid of three groups of patients. Box plots show the Validation of levels of TNF, TGF-beta-1, VEGFA and CASP-8 in FBG, BBG and CBG. *P < 0.05, **P < 0.01.

Combined with the analysis of differential expression of inflammatory cytokines in the three groups of blister fluid, we found that significantly different cytokines were enriched in these biological processes: connective tissue replacement involved in inflammatory response wound healing, regulation of establishment of endothelial barrier, positive regulation of vascular permeability and macrophage differentiation. The closely related inflammatory cytokines include TGF-beta-1, CASP-8, TNF and VEGFA. We further carried out ELIISA verification for these cytokines. ELISA results showed that the expression levels of the above inflammatory cytokines in each group were consistent with the results of proteomics. The expression patterns of TGF-beta-1、TNF and VEGFA were both low CBG, medium BBG, high FBG. But the expression patterns of CASP-8 was low BBG, low CBG, high FBG (**Figure 6**).

## 4 Discussion

Acute fracture injuries are often accompanied by tension-type blisters which can cause longer preoperative waiting times and an increased chance of infection (**1**). Blister fluid can also be considered a form of body fluid created in response to injury and thus may serve as an effective bioindicator for trauma. Recently, blister fluid has become a viable matrix in burn studies as it has been shown to reflect systemic and local immune responses while simultaneously allowing assessment of burn extent and depth (7, 10, 11). Similarly, we hypothesize that blister fluid from patients with FBs can be used as a matrix to reflect systemic inflammation. However, the difference between fracture blisters and blisters caused by other pathogenic factors such as burn blisters and cupping blisters has not been fully explained. Thus, in this study, our aim is to explore the inflammatory landscape of blisters caused by different factors and to provide a preliminary proteomic reference for exploring the relationship between fracture blister formation and trauma.

In this study, we compared 92 inflammation-related proteins across three types of blister fluids: fracture, burn, and cupping. PCA of the 92 inflammatory proteins showed all three groups exhibited distinct but overlapping expressions. In terms of differences, TSLP was uniquely expressed in FBs. Thymus interstitial lymphopoietin (TSLP) is a cytokine similar to interleukin 7 (IL-7). In addition to promoting B cell and Dendritic Cell (DC) activation, TSLP also promotes TH2 cytokine-related inflammation by directly promoting the effector function of CD4+ Th2 cells, basophils, and other granulocyte populations, and at the same time limits DC-derived expression of proinflammatory cytokines and promotes regulatory T cell responses (12). Previous studies have shown that TSLP plays an important role in atopic dermatitis (13). Here we identified a potential role of TSLP in FB fluid for the first time. But whether the occurrence and development of FBs is related to barrier immunity regulated by TSLP still needs further exploration.

In terms of causative factors, fracture blisters and burn blisters tend to be associated with seriously trauma compared to cupping blisters which are associated with little or no trauma. Comparing the protein expression levels across the three groups showed that differentially expressed proteins generally fell into two expression patterns. One where there was high expression in fracture and burn but low expression in cupping, and one where there was high expression in fracture, medium expression in burn, and low expression in cupping. These patterns in expression are likely related to the unique mechanism of each injury and the degree of traumatic stress.

Local inflammatory response pathways play an important role in both patients with fractures and burns through the release of various cytokines (14, 15). Many cytokines, including inflammatory mediators, are involved in multiple steps of the systemic inflammatory response to trauma or severe burns, as well as in the subsequent promotion of wound healing and injury repair processes. Here, IL-6, IL8, TNF-α, CCL20, and IL-22 were overexpressed in the fracture and burn groups compared to the cupping group. Previously, the presence of inflammatory cytokines like IL-6 and CCL20 in saliva fluid led to increased wound healing (16). CCL20 is mainly derived from keratin-forming cells in injured skin (17) where TNF-α plays an important role in its upregulation (18). Furthermore, cell separation due to irritation or injury, such as scratching, can stimulate CCL20 production in keratinocytes (19). Therefore, CCL20 could act as an alarm signal during epidermal injury. Similarly, skin injury may induce co-upregulation of CCL20 with IL8. IL8 causes neutrophils to migrate to the site of injury while CCL20 induces recruitment of CCR6+ Th17 cells, which produce IL-22 that further induces keratinocyte proliferation and may accelerate wound healing (20, 21). The expression of these inflammatory factors is active in fracture and BBs but not cupping blisters, suggesting that fractures and burns provide greater traumatic stimulation and stimulate a more active repair and healing process. This also demonstrates that FB fluid can reflect the state of the systemic internal environment in patients with acute compartment syndrome, just as burn blister fluid can reflect the stress state of burn patients.

During the burn-induced inflammatory response, pro-inflammatory cytokines such as IL-6, IL-8, tumor TNF-α, and anti-inflammatory cytokines such as IL-10 are released. In a study by Hyun Soo Kim (22), it was suggested that the elevation of the above factors after burn injury is caused by interactions within a complex network of cytokines rather than a single factor. These cytokines may be mediators induced by burns or markers of systemic inflammation. IL-6, IL-8, TNF-α, and IL-10 showed high levels of expression in the fracture and burn blister fluid in our study and were significantly different from the blisters in the cupping group. We believe that the intensity of stress caused by trauma is an important factor causing the significant decrease in inflammatory protein expression in cupping blisters compared with the other two groups.

Clinically, the difference between the three types of blisters in terms of concomitant symptoms is pain. In our clinical observations of patients with the three types of blisters, we compared VAS scores between all three groups. Patients in the fracture and burn groups did not have significantly different VAS scores. However, both groups had significantly higher levels of pain compared to the cupping group. Recently, growing evidence suggests typical pro-inflammatory cytokines like IL-6 and TNF-a are highly involved in the development of neuropathic pain (23, 24), while inflammatory chemokines like CCL3 are associated with traditional pain (25). This is important as traumatic injuries such as fractures and burns are caused by a mix of pain sensations including neuropathic pain (26). For example, Ning Zhang et al. (27) found that CCL3 sensitizes TRPV1-mediated signaling; in other words, inflammatory signaling increases the sensitivity of nociceptive receptors and enhances pain perception. In our study, CCL3 was significantly elevated in fracture and BBs, suggesting that CCL3 levels may reflect pain severity.

However, it is important to note that although both fractures and burns are caused by intense traumatic stress and express equally high levels of inflammatory proteins in their blister fluid, the expression of inflammatory proteins in FBs and BBs is not identical. We performed further differential expression analysis between FBG and BBG and found that there were still significant differences in the expression of some proteins. Clinically, fracture patients experience high traumatic stress and ACS, with subsequent secondary hemorrhage in the fracture area, causing excessive tissue swelling. In burn patients, only the skin surface and subcutaneous tissues are subjected to direct physical stress such as flame, and the internal bone remains undamaged. Indeed, less swelling was observed in the injured area of BBG than in FBG. Thus, the main differentiator of fracture and burn injuries is damage to the bone which is often accompanied by the release and change of many associated proteins.

Several fracture related proteins were overexpressed in FBG compared to BBG including osteoprotegerin (OPG) and leukemia inhibitory factor (LIF). In bone tissue, bone resorption by osteoclasts and bone formation by osteoblasts are constantly repeated, thus maintaining a dynamic balance of bone mass. Osteoprotegerin (OPG), a soluble RANKL decoy receptor produced mainly by osteoblasts, inhibits osteoclast formation and proliferation by inhibiting RANKL-RANKL receptor interactions (28, 29). Indeed, previously OPG-deficient mice exhibited severe osteoporosis and even fractures (30, 31). Traditionally, the mechanism of bone remodeling is the activation of transforming growth factor (TGF-β) in the bone matrix by osteoclasts and the activation of osteoblasts. In this study, both OPG and TGF-β were overexpressed in FBG compared to BBG. On one hand, this indicates that osteogenesis and bone remodeling can occur simultaneously at the early stage of fracture in FBs; on the other hand, it indicates that FB fluid as a body fluid is relatively consistent with the microenvironment of blood, and the two are closely linked to some extent. This similarity between FB fluid and blood provides a basis for further investigation into the mechanism of FB formation.

Leukemia inhibitory factor (LIF) is a pleiotropic cytokine released after tissue injury (32). Studies have shown that osteoclast-derived leukemia inhibitory factor (LIF) reduces the expression of sclerostin in osteoblasts and promotes bone formation. Furthermore, during bone resorption, the bone matrix releases transforming growth factor β (TGF-β), which induces osteoblast precursor cells to migrate to the site of bone resorption and promotes bone formation (33, 34). This is as important as previous studies suggest TGF -β induces LIF expression and regulates the migration of osteoblast progenitor cells to restore resorption and promote bone formation (35). Our study is consistent with the findings above. Compared to BBs, LIF and TGF-β were highly expressed in FB fluid. This may be due to TGF -β induced expression of LIF during the fracture repair process. Another explanation may be that fracture misalignment leads to soft tissue lacerations of muscle around the fracture area. This would be more similar to a cutaneous muscle excision injury which leads to elevated LIF expression (36) that is then reflected in blister fluid.

Finally, correlation analysis of proteins in FBG combined with PPI network analysis revealed a key protein module in FB fluid: IL-6, LIF, HGF, and OSM. Critically, these 4 proteins are all STAT3-upregulated extracellular proteins. Studies have found that the activation of STAT3 signaling pathway is associated with skeletal muscle atrophy during burn, cancer and degenerative muscle diseases. Inhibition of STAT3 signaling during burns reduces muscle atrophy (37). But at the same time oxidative stress usually activates the STAT3 signaling pathway, which leads to cell proliferation, survival, differentiation, and angiogenesis (38). We found elevated expression levels of these proteins in FB fluid, and their expression levels showed significant intercorrelation. Combined with the expressions of these factors in other groups, the expression levels of IL-6, LIF and OSM in fracture blisters and burn blisters were significantly higher than those in cupping group. We speculate that the STAT3 signaling pathway may also play a similar role in the formation of fracture blisters. However, whether activation of the STAT3 signaling pathway plays an important role in the occurrence and development of FBs still requires further investigation.

We paid special attention to the differences in expression of TNF, TGF-beta-1, VEGFA and CASP-8 among the three groups. Validation of these cytokines using ELISA was consistent with our results from olink proteomics. Differences in these cytokines may indicate different body responses due to different injury mechanism. Although the results are presented in the form of skin blisters, their internal mechanisms and development outcomes are different. VEGFA and TGF-beta-1 were closely related to positive regulation of vascular permeability, and their expression patterns were low CBG, medium BBG and high FBG in the three groups. This may indicate that the vascular permeability of patients has been actively positively regulated during the occurrence of fracture blisters, which may also be an important factor in the occurrence of fracture blisters. In addition, the expression pattern of TNF in the three groups was the same as above and it participated in the regulation of establishment of endothelial barrier together with VEGFA. TGF-beta-1 was also involved in connective tissue replacement involved in inflammatory response wound healing. This suggested that fracture blisters are also active in the post-injury repair process as a skin injury. In clinical, we also found that fracture blisters often do not leave sequelae of the skin, such as scars and skin fibrosis, which may be related to the active repairment. CASP-8, CSF-1, TGF-beta-1 and VEGFA participate in the regulation of macrophage differentiation. Interestingly, in both proteomics and ELISA results, we found that CASP-8 was highly expressed in FBG, but BBG and CBG were at a low expression level. As a key intermediary molecule in the apoptotic and necrotic pathways, Caspase-8 prevents the formation of necrotic bodies and drives cell apoptosis rather than necrosis after cells receive the stimulation of death signals (39).This may indicate a more active regulation of apoptosis and necrosis in fracture blisters, although further studies are needed to confirm this.

The limitation of our study is that it is an observational study on the levels of cytokines in the fluid of patients with FBs. Although the characteristics of the occurrence and development of FBs have been described at the level of inflammatory cytokines, relevant conclusions about the mechanism of FBs cannot be drawn. Further well-designed prospective studies are needed to elucidate the underlying mechanisms and implications of the changes in cytokine patterns observed in this study.

## 5 Conclusion

In summary, by analyzing the above differentially expressed proteins in the three groups of blister fluids, we suggest the heterogeneity of protein expression in different blister fluids is the result of multiple interacting factors. The type of injury, clinical symptoms, and the degree of traumatic stress likely jointly influence the expression of inflammatory proteins in the blister fluid. More importantly, we identified blister fluid in fractures with ACS as a suitable matrix to reflect the overall internal environment of the body. This provides a basis for further research on the different inflammatory processes of blister induced by different trauma mechanisms and also provides a preliminary reference for studying the mechanism of fracture blister formation.

## Data availability statement

The original contributions presented in the study are included in the article/**Supplementary Material**. Further inquiries can be directed to the corresponding authors.

## Ethics statement

The studies involving human participants were reviewed and approved by Regional Ethics Committee of the Third Hospital of Hebei Medical University. The patients/participants provided their written informed consent to participate in this study.

## Author contributions

ZH and LW contributed to conception and design of the study. ZH provided support for the research and supervised the whole process. YLo and SY collected the biological samples of studied individuals. JLG and QD were involved in the records review and data acquisition. TW and JFG performed data quality control of data. YLo, YLi, and AN performed the statistical analysis and drafted the manuscript. LW critically revised the manuscript and approved the final version. All authors contributed to the article and approved the submitted version.
