# Supplementary material for: Inflammation-related proteomics demonstrate landscape of fracture blister fluid in patients with acute compartment syndrome
Source: Front Immunol. 2023 Apr 6;14:1161479. doi: 10.3389/fimmu.2023.1161479 (PMC10115951; doi:10.3389/fimmu.2023.1161479)
Supplement: Supplementary file 1 [file Table_1.docx]

**Supplementary Table 1. Differential Protein Expression Analysis**

Grey highlighting indicates significance with P-value < 0.01

|  |  |  |  |  | **FBG vs. CBG** | | **FBG vs. BBG** | | **BBG vs. CBG** | |
| --- | --- | --- | --- | --- | --- | --- | --- | --- | --- | --- |
| **Protein** | **Fracture** | **Burn** | **Cupping** | **ANOVA P-value** | **T-value** | **P-value** | **T-value** | **P-value** | **T-value** | **P-value** |
| IL6 | 13.05 | 11.91 | 2.79 | 0.0000 | 26.31 | 0.0000 | 2.07 | 0.0546 | 18.75 | 0.0000 |
| IL-20 | 8.19 | 4.02 | 1.56 | 0.0000 | 29.24 | 0.0000 | 7.96 | 0.0000 | 4.54 | 0.0010 |
| IL10 | 6.74 | 7.24 | 2.61 | 0.0000 | 9.39 | 0.0000 | -1.25 | 0.2383 | 8.46 | 0.0000 |
| VEGFA | 14.29 | 11.96 | 9.72 | 0.0000 | 12.32 | 0.0000 | 4.98 | 0.0006 | 3.99 | 0.0014 |
| OPG | 11.43 | 9.74 | 8.39 | 0.0000 | 9.86 | 0.0000 | 5.98 | 0.0000 | 4.03 | 0.0014 |
| IL8 | 11.80 | 11.25 | 4.33 | 0.0000 | 15.88 | 0.0000 | 0.60 | 0.5633 | 7.28 | 0.0000 |
| CCL20 | 13.73 | 11.37 | 6.55 | 0.0000 | 11.62 | 0.0000 | 2.66 | 0.0284 | 4.49 | 0.0006 |
| LAP TGF-beta-1 | 7.80 | 6.68 | 5.64 | 0.0000 | 6.52 | 0.0002 | 7.41 | 0.0000 | 3.16 | 0.0150 |
| MCP-1 | 15.51 | 15.08 | 12.14 | 0.0000 | 6.06 | 0.0009 | 1.52 | 0.1650 | 4.76 | 0.0010 |
| LIF | 8.35 | 5.40 | 2.52 | 0.0000 | 11.05 | 0.0000 | 4.29 | 0.0005 | 4.44 | 0.0007 |
| IL-24 | 6.83 | 4.20 | 1.25 | 0.0000 | 12.93 | 0.0000 | 3.65 | 0.0035 | 4.12 | 0.0017 |
| CCL19 | 12.16 | 8.14 | 8.32 | 0.0000 | 7.09 | 0.0003 | 5.60 | 0.0005 | -0.20 | 0.8469 |
| CSF-1 | 10.79 | 9.65 | 8.79 | 0.0000 | 4.99 | 0.0018 | 8.79 | 0.0000 | 2.14 | 0.0714 |
| MCP-3 | 7.44 | 8.16 | 1.55 | 0.0000 | 13.54 | 0.0000 | -0.63 | 0.5424 | 5.89 | 0.0003 |
| MMP-1 | 15.29 | 14.26 | 10.69 | 0.0000 | 9.10 | 0.0000 | 1.40 | 0.1873 | 4.62 | 0.0005 |
| OSM | 6.71 | 8.39 | 2.83 | 0.0000 | 6.23 | 0.0000 | -2.10 | 0.0565 | 6.46 | 0.0000 |
| IL-1 alpha | 1.82 | 4.29 | 6.58 | 0.0000 | -5.55 | 0.0003 | -4.48 | 0.0002 | -2.76 | 0.0229 |
| CD40 | 12.25 | 11.86 | 10.45 | 0.0000 | 5.44 | 0.0009 | 1.55 | 0.1518 | 3.55 | 0.0041 |
| FGF-5 | 2.27 | 1.59 | 1.30 | 0.0000 | 6.59 | 0.0000 | 5.12 | 0.0001 | 3.02 | 0.0126 |
| uPA | 12.21 | 10.52 | 10.47 | 0.0000 | 4.63 | 0.0013 | 5.29 | 0.0001 | 0.12 | 0.9071 |
| CXCL1 | 11.57 | 10.73 | 7.02 | 0.0000 | 7.06 | 0.0000 | 1.05 | 0.3113 | 4.35 | 0.0007 |
| PD-L1 | 5.92 | 4.91 | 4.07 | 0.0000 | 5.21 | 0.0002 | 4.05 | 0.0006 | 2.67 | 0.0269 |
| MCP-2 | 11.07 | 9.54 | 8.13 | 0.0000 | 6.63 | 0.0000 | 2.74 | 0.0190 | 2.21 | 0.0449 |
| NT-3 | 3.19 | 3.05 | 1.61 | 0.0000 | 6.33 | 0.0001 | 0.46 | 0.6551 | 4.04 | 0.0012 |
| LIF-R | 2.54 | 2.73 | 2.05 | 0.0000 | 4.75 | 0.0005 | -1.69 | 0.1121 | 5.37 | 0.0001 |
| SCF | 9.20 | 8.70 | 7.99 | 0.0001 | 3.52 | 0.0098 | 3.56 | 0.0021 | 2.07 | 0.0766 |
| IL-15RA | 3.16 | 2.22 | 2.06 | 0.0001 | 3.61 | 0.0046 | 5.39 | 0.0000 | 0.55 | 0.5976 |
| CXCL5 | 10.68 | 9.84 | 5.77 | 0.0001 | 8.09 | 0.0000 | 0.76 | 0.4622 | 3.80 | 0.0034 |
| TNFRSF9 | 6.42 | 5.63 | 5.19 | 0.0001 | 4.05 | 0.0029 | 3.75 | 0.0015 | 1.38 | 0.1971 |
| IL-22 RA1 | 3.77 | 4.54 | 2.68 | 0.0001 | 2.94 | 0.0144 | -2.66 | 0.0160 | 4.77 | 0.0006 |
| IL-18R1 | 7.93 | 8.60 | 6.91 | 0.0002 | 2.77 | 0.0197 | -2.78 | 0.0111 | 4.74 | 0.0011 |
| ARTN | 2.85 | 1.92 | 1.19 | 0.0002 | 8.28 | 0.0000 | 2.22 | 0.0484 | 1.81 | 0.1016 |
| TNF | 5.96 | 4.39 | 3.54 | 0.0002 | 5.30 | 0.0001 | 2.88 | 0.0111 | 1.53 | 0.1497 |
| CDCP1 | 8.72 | 8.38 | 6.90 | 0.0003 | 3.22 | 0.0144 | 1.41 | 0.1751 | 2.59 | 0.0347 |
| CD5 | 6.66 | 7.06 | 5.40 | 0.0004 | 4.39 | 0.0003 | -1.23 | 0.2323 | 5.62 | 0.0001 |
| TGF-alpha | 6.60 | 5.49 | 4.68 | 0.0005 | 4.31 | 0.0025 | 2.41 | 0.0353 | 1.37 | 0.1912 |
| CD244 | 5.44 | 5.43 | 4.71 | 0.0006 | 3.17 | 0.0166 | 0.11 | 0.9118 | 2.73 | 0.0210 |
| CD6 | 4.92 | 4.89 | 3.94 | 0.0007 | 3.50 | 0.0057 | 0.20 | 0.8419 | 3.53 | 0.0070 |
| TSLP | 1.79 | 1.47 | 1.02 | 0.0009 | 4.51 | 0.0009 | 1.82 | 0.0911 | 2.13 | 0.0513 |
| IL-17C | 7.30 | 4.62 | 5.36 | 0.0013 | 2.52 | 0.0334 | 3.68 | 0.0030 | -0.77 | 0.4526 |
| TNFSF14 | 4.09 | 6.23 | 3.98 | 0.0014 | 0.33 | 0.7475 | -2.97 | 0.0127 | 3.29 | 0.0093 |
| CXCL9 | 8.61 | 7.35 | 7.42 | 0.0015 | 2.70 | 0.0262 | 3.62 | 0.0030 | -0.14 | 0.8892 |
| MCP-4 | 14.52 | 12.39 | 14.29 | 0.0019 | 0.27 | 0.7981 | 5.20 | 0.0001 | -2.11 | 0.0684 |
| CASP-8 | 7.13 | 5.00 | 5.41 | 0.0021 | 4.35 | 0.0006 | 2.80 | 0.0185 | -0.53 | 0.6071 |
| 4E-BP1 | 10.76 | 9.94 | 11.33 | 0.0027 | -3.89 | 0.0028 | 1.87 | 0.0957 | -3.10 | 0.0122 |
| SLAMF1 | 2.37 | 2.34 | 1.99 | 0.0039 | 3.35 | 0.0057 | 0.26 | 0.8009 | 3.16 | 0.0089 |
| IL-10RB | 6.61 | 6.69 | 5.98 | 0.0077 | 2.09 | 0.0752 | -0.60 | 0.5575 | 2.30 | 0.0521 |
| CXCL6 | 7.72 | 7.14 | 6.23 | 0.0091 | 3.30 | 0.0061 | 1.43 | 0.1688 | 1.93 | 0.0767 |
| IL7 | 2.25 | 2.54 | 3.17 | 0.0098 | -2.57 | 0.0335 | -1.35 | 0.1945 | -1.69 | 0.1253 |
| TWEAK | 7.98 | 9.54 | 9.06 | 0.0106 | -1.93 | 0.0850 | -2.98 | 0.0107 | 0.71 | 0.4899 |
| CCL3 | 8.25 | 8.49 | 5.44 | 0.0126 | 4.68 | 0.0002 | -0.21 | 0.8359 | 2.78 | 0.0190 |
| IL-10RA | 1.59 | 1.63 | 1.38 | 0.0202 | 2.23 | 0.0558 | -0.55 | 0.5924 | 2.32 | 0.0392 |
| CST5 | 5.46 | 5.30 | 4.92 | 0.0290 | 1.93 | 0.0967 | 1.23 | 0.2414 | 1.29 | 0.2332 |
| CCL4 | 7.12 | 6.92 | 4.77 | 0.0316 | 4.16 | 0.0006 | 0.20 | 0.8436 | 2.23 | 0.0486 |
| IL-2RB | 1.52 | 1.61 | 1.33 | 0.0366 | 2.35 | 0.0316 | -0.98 | 0.3428 | 2.97 | 0.0103 |
| CCL23 | 10.28 | 9.95 | 9.28 | 0.0480 | 1.78 | 0.1158 | 1.46 | 0.1587 | 1.22 | 0.2613 |
| Flt3L | 10.89 | 9.81 | 10.25 | 0.0517 | 0.98 | 0.3590 | 3.50 | 0.0024 | -0.65 | 0.5334 |
| IL33 | 3.95 | 4.40 | 2.86 | 0.0543 | 1.91 | 0.0831 | -0.87 | 0.3976 | 2.39 | 0.0324 |
| Beta-NGF | -0.30 | -0.43 | -0.61 | 0.0548 | 2.67 | 0.0153 | 1.32 | 0.2011 | 2.09 | 0.0637 |
| CCL28 | 2.82 | 2.29 | 2.91 | 0.0610 | -0.31 | 0.7648 | 2.89 | 0.0097 | -2.33 | 0.0535 |
| TNFB | 3.53 | 3.59 | 4.24 | 0.0616 | -2.25 | 0.0489 | -0.22 | 0.8274 | -1.81 | 0.0947 |
| NRTN | 1.82 | 1.82 | 1.44 | 0.0617 | 2.83 | 0.0104 | 0.01 | 0.9891 | 2.81 | 0.0143 |
| IL18 | 9.56 | 10.08 | 10.66 | 0.0662 | -2.47 | 0.0305 | -1.18 | 0.2572 | -1.12 | 0.2840 |
| CXCL10 | 10.87 | 9.40 | 9.92 | 0.0699 | 1.26 | 0.2492 | 2.13 | 0.0601 | -0.53 | 0.6022 |
| EN-RAGE | 5.95 | 5.78 | 3.89 | 0.0760 | 3.01 | 0.0075 | 0.19 | 0.8557 | 2.06 | 0.0614 |
| CD8A | 5.70 | 6.24 | 7.16 | 0.0795 | -2.00 | 0.0804 | -0.97 | 0.3478 | -1.12 | 0.2871 |
| IFN-gamma | 4.76 | 5.64 | 4.70 | 0.1002 | 0.11 | 0.9175 | -2.15 | 0.0466 | 1.69 | 0.1167 |
| FGF-23 | 1.44 | 1.15 | 0.77 | 0.1077 | 2.71 | 0.0135 | 0.99 | 0.3325 | 1.60 | 0.1336 |
| FGF-21 | 4.74 | 5.64 | 4.14 | 0.1229 | 0.94 | 0.3638 | -1.46 | 0.1638 | 2.10 | 0.0544 |
| DNER | 7.59 | 7.62 | 7.11 | 0.1244 | 1.42 | 0.1970 | -0.19 | 0.8523 | 1.47 | 0.1789 |
| HGF | 10.81 | 10.62 | 9.94 | 0.1467 | 1.80 | 0.0995 | 0.55 | 0.5890 | 1.47 | 0.1737 |
| AXIN1 | 5.61 | 4.66 | 5.40 | 0.1608 | 0.31 | 0.7647 | 2.15 | 0.0493 | -1.03 | 0.3277 |
| IL2 | 1.32 | 1.41 | 1.25 | 0.1699 | 0.83 | 0.4300 | -1.23 | 0.2419 | 1.57 | 0.1392 |
| CCL25 | 5.83 | 5.05 | 5.13 | 0.1837 | 1.34 | 0.2175 | 1.53 | 0.1546 | -0.12 | 0.9069 |
| STAMBP | 7.90 | 7.55 | 8.50 | 0.2345 | -1.18 | 0.2618 | 0.78 | 0.4482 | -1.71 | 0.1121 |
| CXCL11 | 5.28 | 4.99 | 4.62 | 0.2472 | 1.32 | 0.2324 | 0.81 | 0.4400 | 0.62 | 0.5503 |
| TRANCE | 3.29 | 3.62 | 4.00 | 0.2505 | -1.50 | 0.1646 | -0.91 | 0.3765 | -0.75 | 0.4675 |
| GDNF | 2.95 | 2.72 | 2.43 | 0.2665 | 1.51 | 0.1583 | 0.87 | 0.3938 | 0.84 | 0.4196 |
| IL4 | 1.61 | 1.35 | 1.57 | 0.2716 | 0.23 | 0.8226 | 1.74 | 0.0962 | -1.40 | 0.1876 |
| FGF-19 | 7.46 | 7.89 | 7.88 | 0.2726 | -1.09 | 0.3049 | -1.51 | 0.1534 | 0.03 | 0.9803 |
| IL-17A | 3.19 | 3.31 | 2.52 | 0.3009 | 1.96 | 0.0772 | -0.22 | 0.8332 | 1.27 | 0.2297 |
| IL-12B | 6.59 | 6.50 | 6.91 | 0.3728 | -1.08 | 0.3109 | 0.36 | 0.7220 | -1.18 | 0.2615 |
| IL13 | 0.69 | 0.79 | 0.62 | 0.4520 | 0.69 | 0.5011 | -0.80 | 0.4385 | 1.33 | 0.2065 |
| IL-20RA | 2.91 | 2.72 | 3.10 | 0.4562 | -0.69 | 0.5077 | 0.71 | 0.4920 | -1.11 | 0.2843 |
| MMP-10 | 9.22 | 9.16 | 8.72 | 0.5681 | 1.18 | 0.2583 | 0.13 | 0.8990 | 0.84 | 0.4124 |
| ST1A1 | 3.39 | 3.35 | 2.75 | 0.6282 | 1.21 | 0.2405 | 0.06 | 0.9503 | 0.95 | 0.3613 |
| TRAIL | 6.12 | 6.33 | 6.40 | 0.6428 | -0.63 | 0.5494 | -0.80 | 0.4372 | -0.15 | 0.8875 |
| IL5 | 1.43 | 1.50 | 1.38 | 0.6442 | 0.44 | 0.6684 | -0.62 | 0.5456 | 0.91 | 0.3792 |
| SIRT2 | 7.68 | 7.25 | 7.77 | 0.6496 | -0.15 | 0.8799 | 0.77 | 0.4544 | -0.79 | 0.4437 |
| ADA | 6.00 | 6.36 | 6.27 | 0.7042 | -0.56 | 0.5858 | -0.89 | 0.3854 | 0.19 | 0.8524 |
| CX3CL1 | 2.98 | 2.83 | 2.79 | 0.7196 | 0.54 | 0.6066 | 0.78 | 0.4453 | 0.11 | 0.9114 |
| CCL11 | 5.94 | 5.66 | 5.84 | 0.7239 | 0.20 | 0.8506 | 0.99 | 0.3391 | -0.34 | 0.7443 |
